# Tissue Sources Influence the Morphological and Morphometric Characteristics of Collagen Membranes for Guided Bone Regeneration

**DOI:** 10.3390/polym16243499

**Published:** 2024-12-16

**Authors:** Josefa Alarcón-Apablaza, Karina Godoy-Sánchez, Marcela Jarpa-Parra, Karla Garrido-Miranda, Ramón Fuentes

**Affiliations:** 1 Doctoral Program in Morphological Sciences, Faculty of Medicine, Universidad de La Frontera, Temuco 4780000, Chile; josefa.alarcon@ufrontera.cl; 2 Research Center in Dental Sciences (CICO-UFRO), Dental School, Faculty of Dentistry, Universidad de La Frontera, Temuco 4780000, Chile; 3 Scientific and Technological Bioresource Nucleus (BIOREN-UFRO), Universidad de La Frontera, Temuco 4780000, Chile; karina.godoy@ufrontera.cl (K.G.-S.); karla.garrido@ufrontera.cl (K.G.-M.); 4 Natural Resources and Polymers Research Laboratory, Universidad Adventista de Chile, Chillán 3780000, Chile; marcelajarpa@unach.cl; 5 Department of Integral Adult Dentistry, Dental School, Faculty of Dentistry, Universidad de La Frontera, Temuco 4780000, Chile

**Keywords:** biopolymers, biomaterials, natural polymers, biocompatible materials, barrier membrane, bone regeneration, GBR, guided tissue regeneration, physicochemical characterization

## Abstract

(1) Background: Collagen, a natural polymer, is widely used in the fabrication of membranes for guided bone regeneration (GBR). These membranes are sourced from various tissues, such as skin, pericardium, peritoneum, and tendons, which exhibit differences in regenerative outcomes. Therefore, this study aimed to evaluate the morphological and chemical properties of porcine collagen membranes from five different tissue sources: skin, pericardium, dermis, tendons, and peritoneum. (2) Methods: The membrane structure was analyzed using energy-dispersive X-ray spectrometry (EDX), variable pressure scanning electron microscopy (VP-SEM), Fourier transform infrared spectroscopy (FTIR), and thermal stability via thermogravimetric analysis (TGA). The absorption capacity of the membranes for GBR was also assessed using an analytical digital balance. (3) Results: The membranes displayed distinct microstructural features. Skin- and tendon-derived membranes had rough surfaces with nanopores (1.44 ± 1.24 µm and 0.46 ± 0.1 µm, respectively), while pericardium- and dermis-derived membranes exhibited rough surfaces with macropores (78.90 ± 75.89 µm and 64.89 ± 13.15 µm, respectively). The peritoneum-derived membrane featured a rough surface of compact longitudinal fibers with irregular macropores (9.02 ± 3.70 µm). The thickness varied significantly among the membranes, showing differences in absorption capacity. The pericardium membrane exhibited the highest absorption, increasing by more than 10 times its initial mass. In contrast, the skin-derived membrane demonstrated the lowest absorption, increasing by less than 4 times its initial mass. Chemical analysis revealed that all membranes were primarily composed of carbon, nitrogen, and oxygen. Thermogravimetric and differential scanning calorimetry analyses showed no significant compositional differences among the membranes. FTIR spectra confirmed the presence of collagen, with characteristic peaks corresponding to Amide A, B, I, II, and III. (4) Conclusions: The tissue origin of collagen membranes significantly influences their morphological characteristics, which may, in turn, affect their osteogenic properties. These findings provide valuable insights into the selection of collagen membranes for GBR applications.

## 1. Introduction

Guided bone regeneration (GBR) is an essential surgical procedure in dentistry and maxillofacial surgery, the main objective of which is to generate an ideal condition for reconstructing critical bone defects in dental implants, periodontal defects, and post-extraction sockets [1,2,3]. For bone regeneration to be successful, the bone defect must be isolated from the surrounding soft tissues. This process may require a healing period of at least 16 to 24 weeks. During this time, it is important to protect the treated area to optimize the bone regeneration outcomes [1,4]. It is essential to use a barrier membrane to isolate the bone defect and perform GBR techniques [5,6]. The main function of barrier membranes is to guide soft and hard tissue regeneration by preventing the growth of epithelium and connective tissue in the bone compartment [5,6]. The indication for GBR is determined by the type and size of the remaining bone wall. It is especially recommended in cases of large defects or loss of the bone wall [6].

Since barrier membranes play a pivotal role in GBR, they must fulfill specific characteristics to ensure adequate bone graft support, protect the site from invasion of unwanted tissue, and promote new bone formation without external interference [5,6]. Ideal membrane characteristics, apart from the barrier effect, include (a) biocompatibility, (b) biological activity, (c) porosity/occlusive properties, (d) mechanical properties, (e) exposure tolerance, and (f) biodegradability [7,8,9,10,11]. In addition, the detection of water absorption properties is essential in studies of barrier membranes. Higher water absorption performance enhances the nutritional absorption capacity and the ability to absorb exudates in a GTR membrane, which in turn promotes cell growth and the absorption of inflammatory exudates [12].

Membranes used in guided bone regeneration (GBR) are developed from various materials, mainly classified into synthetic polymers, natural polymers, metals, and inorganic compounds [5,9]. These materials can be biodegradable or non-biodegradable [13,14]. Non-resorbable membranes, such as polytetrafluoroethylene (PTFE) in its expanded form (e-PTFE) or titanium, constituted the first generation of barrier membranes. In general, these types of membranes are used due to their high strength and ability to integrate into bone (osseointegration), providing structural support during healing. However, these membranes require surgical removal after tissue regeneration [9,15,16]. In addition, non-biodegradable membranes have been reported to show an increased risk of complications related to membrane exposure during implantation [17]. Subsequently, a second generation of membranes made of resorbable materials was developed, which has been widely used in different clinical situations. Biodegradable materials such as PLGA (polylactic acid-co-glycolic acid) break down in a controlled manner in the body, allowing them to be absorbed as bone regeneration progresses [9,17].

Collagen-based membranes are among the most widely used biodegradable membranes of natural origin for guided bone regeneration (GBR) [9,11,14,18]. Collagen is a major component of the extracellular matrix (ECM) in both hard and soft tissues and is found in various tissues such as bone, teeth, tendons, and skin [19]. The three most common types of collagens are types I, II, and III, which differ in helix size and length. Type I collagen, the most abundant and important, is primarily sourced from skin, tendons, and bone. Type II collagen is extracted from cartilage, and type III is found in the vascular system. Notably, type I collagen accounts for approximately 90% of the body’s collagen and is the most commonly used for various biomedical applications. In the skin, type I collagen constitutes 80–85% of the dermal extracellular matrix (ECM), while type III collagen makes up about 8–11% [20]. Tendons are composed of 70% water and 30% dry mass, of which 60–80% is type I collagen and 2% is elastin. To a lesser extent, types III and V collagen are also present in tendons [21]. The pericardium and peritoneum are collagen type I-rich tissues, which is why they have been widely used in biomaterials across various areas of health sciences [22,23].

Collagen matrices, especially those derived from type I, are widely used as ECM substitutes for tissue regeneration and repair. This is particularly relevant since natural bone is primarily composed of type I collagen [24,25]. These membranes have garnered significant attention due to collagen’s role as a key component of connective tissue, where it facilitates cell–matrix communication. Furthermore, collagen is biocompatible, and its degradation poses no harmful effects on tissues while promoting wound healing [9]. However, one limitation of collagen membranes is their lack of rigidity, which can restrict their ability to maintain space. Despite this, collagen membranes can be effectively used in alveolar bone defects that do not require additional fixation and stability, such as in cases of bone dehiscence and fenestration defects [3,26].

Collagen membranes used in dentistry are mainly derived from tendons, dermis, skin, peritoneum, or pericardium and are commonly of porcine origin [15,27]. The origin and manufacturing process of the membranes will determine their physicochemical properties [27]. They can be created with different techniques. Generally, the collagen fraction is isolated and purified, then precipitated in fibrillar form by changing the ionic strength, pH, or increasing the temperature to 37 °C followed by an air evaporation and lyophilization step [28,29].

Despite the proven efficacy of collagen membranes, differences in their regenerative properties based on tissue origin have been demonstrated [30,31,32]. However, it remains unclear how the specific properties of collagen membranes from different origins affect their bone regeneration capacity. This lack of understanding regarding the differences between various origins of collagens used in guided bone regeneration (GBR) represents a significant gap in current knowledge, highlighting the need to study how the origin of the membranes influences their morphological characteristics and their impact on bone regeneration outcomes. Addressing this information gap could have significant implications for optimizing clinical techniques and improving the quality of outcomes in GBR procedures. Therefore, this study aims to analyze the characteristics of commercially available porcine collagen membranes derived from different tissues (skin, pericardium, dermis, tendons, and peritoneum), obtained from multiple animals, focusing on their morphology and composition to understand the factors that contribute to differences in regenerative outcomes.

## 2. Materials and Methods

Five porcine collagen membranes of different origins for GBR were evaluated using three-dimensional surface analysis by variable pressure scanning electron microscopy (VP-SEM) (Hitachi SU3500, Tokyo, Japan) coupled to a semi-quantitative elemental analysis by energy-dispersive X-Ray spectrometry detector (EDX) (XFlash^®^ Detector 410 and Quantax Esprit 1.8.1 Software controller) (Bruker, Berlin, Germany). In addition, the composition of the membranes was analyzed by Fourier transform infrared spectroscopy (FTIR) (TENSOR 27, Bruker, Munich, Germany) and thermal stability via thermogravimetric analysis (TGA) (Thermogravimetric Analysis TGA/DSC STA 6000, Perkin Elmer, Waltham, MA, USA) digital balance. The membranes used are summarized in Table 1. The specifications of the membranes studied were obtained from the manufacturer’s technical data.

### 2.1. Microstructure and Semi-Quantitative Elemental Microanalysis of Membranes for Guided Bone Regeneration by Variable Pressure Scanning Electron Microscopy (VP-SEM) and Energy-Dispersive X-Ray Spectroscopy (EDX)

#### 2.1.1. Microstructural Analysis

A qualitative three-dimensional morphological analysis of five collagen membranes for GRB was performed using a VP-SEM. The membranes were immediately removed from their original packaging before the three-dimensional surface analysis. Visualization was performed using a chemical contrast detector (Backscatter, BSE) (Hitachi SU3500, Tokyo, Japan) at variable pressure with no other sputtering.

Images of both sides of the membranes were taken to analyze and compare their surfaces under the following parameters: 20.0 kV, 80 Pa, and 9.3–10.7 mm. The qualitative analysis of the surface morphology of membranes of different origins was mainly based on the presence and morphology of pores and surface roughness. Photomicrograph magnifications were standardized to X100. Images were acquired and analyzed with Hitachi software control and the Image J 1.53 k Java 1.8.0_172 software (Wayne Rasband et al., National Institutes of Health, Bethesda, MA, USA).

#### 2.1.2. Microstructural Analysis—Quantitative

After the qualitative analysis, a quantitative three-dimensional morphological analysis of the five collagen membranes for GBR was performed by VP-SEM (Hitachi SU3500, Tokyo, Japan). The quantitative analysis was based on membrane thickness and pore size. The samples were adhered to the sample holder with double-sided carbon tape.

For membrane thickness analysis, the magnifications of the photomicrographs at X30 and X50 were standardized under the following parameters: 20.0 kV, 20 Pa, and 21.2–23.1 mm.

The pore size of the membranes was determined using the ImageJ 1.53 k Java 1.8.0_172 software. The magnifications of the photomicrographs were standardized to X500 and X100, while in cases of specific regions of interest, X1000 and X2000 images were obtained under the following parameters: 20.0 kV, 80 Pa, and 9.3–10.7 mm. An area of the image was chosen randomly, and the threshold was adjusted to the visualized, bright (membrane), and dark (pores) regions; then the cutoff value was recorded in the dark areas. At least six zones of each membrane were measured to analyze thickness uniformity. Two calibrated observers analyzed the morphological characteristics at different magnifications.

#### 2.1.3. Semi-Quantitative Elemental Microanalysis

The elemental semi-quantitative analysis of the various membranes was conducted using EDX with a XFlash^®^ Detector 410 and Quantax Esprit 1.8.1 Software (Bruker, Berlin, Germany), connected to the VP-SEM system. To quantify the weight percentage (wt. %) and elemental distribution of the samples, three analysis points were randomly selected from each region of interest (ROI), ensuring a representative analysis across different areas of the membrane. The wt.% of each chemical element in the sample was determined based on the X-ray spectra obtained at each point. The evaluation was carried out under the following parameters: magnification X250, applied voltage of 20.0 kV, and working distance (WD) between 9.5 and 10.7 mm. These parameters were chosen to achieve an optimal balance between resolution and elemental detection sensitivity, ensuring accurate identification of elements while preventing damage to the sample. As a semi-quantitative method, the analysis provides an estimation of elemental composition within a specific range, in contrast to absolute quantification typically achieved with more advanced techniques.

### 2.2. Absorption Analysis of Membranes for Guided Bone Regeneration Using an Analytical Digital Balance

The absorption capacity of the five collagen membranes for GBR was analyzed with a digital analytical balance (A&D HR-120, Japan). It was performed by hydrating standardized 1 x 1 cm membrane samples with phosphate buffer solution (PBS) at pH 7.4. All membranes were soaked in 1 mL of PBS [1]. Data were recorded every 2 min until adsorption saturation of each membrane. Each sample was kneaded in the dry state and at each time point. The mass increase was standardized to the initial dry mass of the sample. The absorption capacity is expressed as a standardized fold increase.

### 2.3. Thermogravimetric Analysis and Differential Scanning Calorimetry in the Collagen Membranes for GBR

The thermogravimetric analysis was achieved to measure the loss of weight of the collagen membrane as a function of temperature. Approximately 10 ± 3.11 mg of samples were subjected to two thermogravimetric analyses: first, from 25 °C to 850 °C (10 °C per minute rate) to evaluate collagen membranes samples stability in the air atmosphere by monitoring weight loss, and second, differential thermal behavior (DSC) from 25 °C to 850 °C (50 °C per minute rate) (Thermogravimetric Analysis TGA/DSC STA 6000, Perkin Elmer, Waltham, MA, USA).

### 2.4. Functional Groups by Infrared Spectroscopy (FT-IR) in the Collagen Membranes for GBR

The functional groups of the collagen were analyzed by FT-IR. Approximately 5 mg of the collagen membranes were mixed with 50 mg potassium bromide powder (KBr-Merck), finely ground manually with an agate mortar, and pressed into 13 mm discs with a manual press. Disc samples (20 mg) were inserted into the system sample chamber for analysis. The characterization of functional groups was analyzed in the mid-infrared from 4000 to 400 cm^−1^ with an FT-IR spectrometer (TENSOR 27, Bruker, Munich, Germany) equipped with a DLATG detector. Data collection and analysis were realized using spectroscopy software (OPUSTM, Bruker, Munich, Germany).

## 3. Results

### 3.1. Microstructure and Semi-Quantitative Elemental Microanalysis of Membranes for Guided Bone Regeneration by Variable Pressure Scanning Electron Microscopy (VP-SEM) and Energy Dispersive X-Ray Spectroscopy (EDX)

#### 3.1.1. Microstructural Analysis—Qualitative

**Figure 1:**
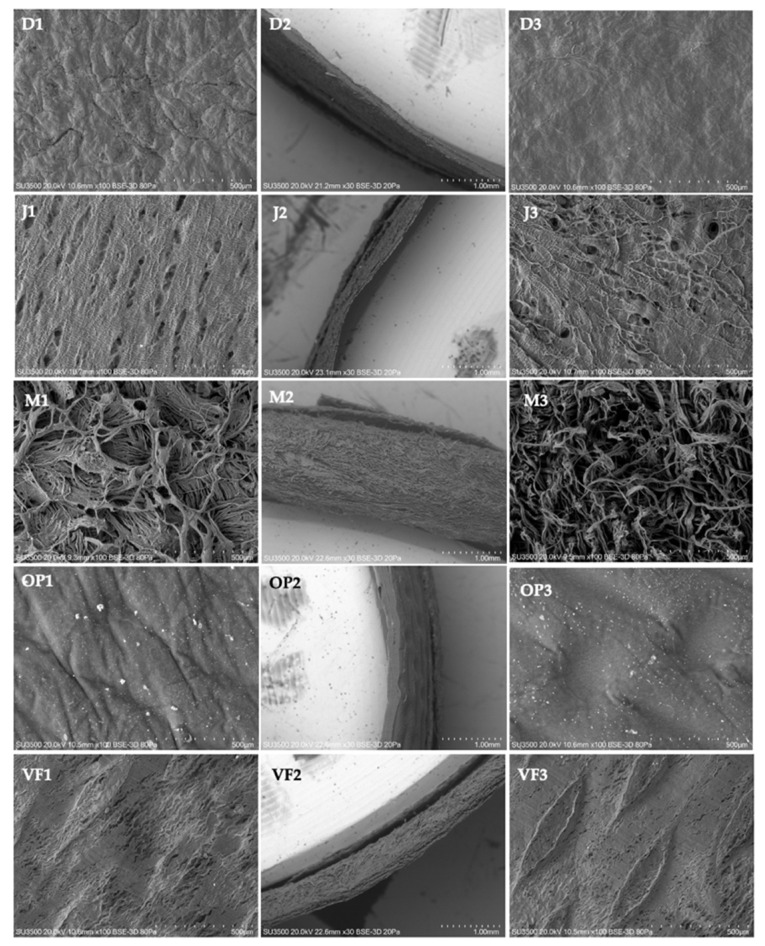
Three-dimensional structures of membranes for guided bone regeneration observed by variable pressure scanning electron microscopy. (D) Diaderm; (J) Jason; (M) Mucoderm; (OP) OSSiX Plus; (VF) Via Flex. (1,3) Membrane surface views; (2) Membrane thickness.

The differences in the morphological characteristics of the membranes for GBR analyzed in this study are shown in Figure 1. The SEM images revealed that the origin of the membranes determines their morphological characteristics, since the membranes had marked differences in the microstructures. Diaderm and OSSiX plus, made from skin and tendons, respectively, presented a rough surface (Figure 1(D1,OP1)) and a smooth opposite surface (Figure 1(D3,OP3)), characterized by the absence of visible pores. Jason membrane created from pericardium exhibited a rough surface with striations and multiple rounded macropores on both surfaces. Jason membrane had a surface with more regular characteristics (Figure 1(J1)) than its antagonist surface (Figure 1(J3)). Mucoderm, manufactured from the dermis, presented great roughness and the presence of a network of fibers that form a highly porous surface (Figure 1(M1,M3)). Via Flex, manufactured from peritoneum, had a rough surface (Figure 1(VF1,VF3)) formed by compact longitudinal fibers. It was characterized by uniform fusiform elevations and irregular macropores on both surfaces.

#### 3.1.2. Microstructural Analysis—Quantitative

1. Pore Size

The pore size of the membranes analyzed in this study is shown in Table 2. The pore size varies significantly among the membranes. OssiX plus and Diaderm were characterized by the presence of nanopores. OssiX plus exhibited an average pore size of 0.46 µm with a low standard deviation, suggesting consistency in manufacturing and uniformity in pore size. Jason membrane and Diaderm exhibit an average pore size with a large standard deviation, indicating a high variability in pore size. This difference in pore size variability may influence the efficiency of the membranes to allow the passage of nutrients and cells, which could have implications for the ability to promote bone regeneration [18].

2. Membrane Thickness

The membrane thicknesses analyzed in this study are shown in Table 3. When analyzing the data provided on the thickness of collagen membranes for GBR, significant variability is noted among the different membranes evaluated. In contrast, membranes such as mucoderm exhibit a notably higher average thickness of 1352 µm and a relatively low standard deviation of 120 µm, indicating consistency in their manufacture. Others, such as Via Flex, have an average thickness of 311.83 µm with a considerably higher standard deviation of 219.19 µm, suggesting greater variability in thickness. These differences may influence the choice of the appropriate membrane for specific clinical applications.

#### 3.1.3. Semi-Quantitative Elemental Microanalysis

**Figure 2:**
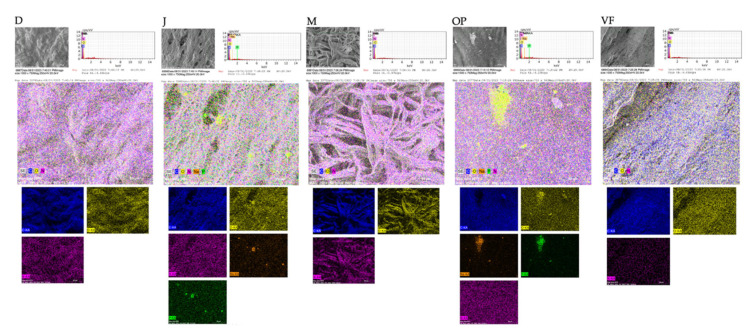
Mapping elemental distribution in membranes: (D) Diaderm. (J) Jason membrane. (M) mucoderm. (V) Via Flex. (OP) OSSiX plus. (VF) Via Flex. (Mag: ×250). Blue = carbon; yellow = oxygen; pink = nitrogen; orange = sodium; green = phosphorus.

The EDX method included element distribution mapping and semi-quantitative elemental microanalysis. Figure 2 provides chemical information on the membrane surfaces through elemental mapping using element-specific colors. A similar composition is observed on the membrane surfaces in all the zones analyzed, composed mainly of carbon, nitrogen, and oxygen. Jason membrane and OSSiX plus showed the presence of sodium and phosphorus in low percentages. Table 4 shows the percentage by weight of the main chemical elements detected in each sample. Carbon (C) was identified as the main element, followed by nitrogen (N) and oxygen (O) in similar percentages.

### 3.2. Absorption of Membranes

The adsorption capacity of the membranes analyzed in this study is shown in Table 5. All membranes showed an increase in mass during hydration. In general, there was an increase in absorption over time, suggesting that the membranes have an absorption capacity that increases with time after application until a saturation point is reached. Diaderm M, Jason membrane, and mucoderm saturated their absorption after 8 min. Ossix plus and Via Flex saturated their absorption after 10 min. Differences in absorption levels were observed among the different membranes. Jason membrane had the highest absorption, followed by OSSIX plus, while Diaderm M had the lowest absorption until stabilization.

### 3.3. Thermogravimetric Analysis and Differential Scanning Calorimetry in the Collagen Membranes for GBR

**Figure 3:**
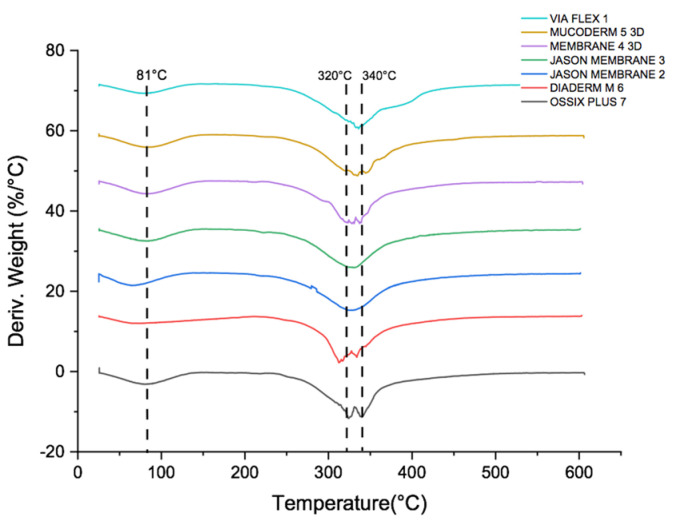
Thermogravimetric analysis TGA-DSC.

Figure 3 shows that there are no differences in composition between the membranes analyzed. Two thermal transitions, or significant mass losses, are observed (Figure 3 and Table 6), which correspond to those reported for collagen: the first, between 50 and 100 °C, is associated with the loss of structural water (H°), and the second, beginning between 200 and 220 °C, corresponds to the decomposition of organic carbon, which is completely consumed by 350 °C, a range associated with the volatilization of organic compounds. In general, the values of humidity, ash, volatiles, and fixed carbon (Table 7) are similar across all the membranes studied, and do not show significant variation.

### 3.4. Functional Groups by Infrared Spectroscopy (FT-IR) in the Collagen Membranes for GBR

**Figure 4:**
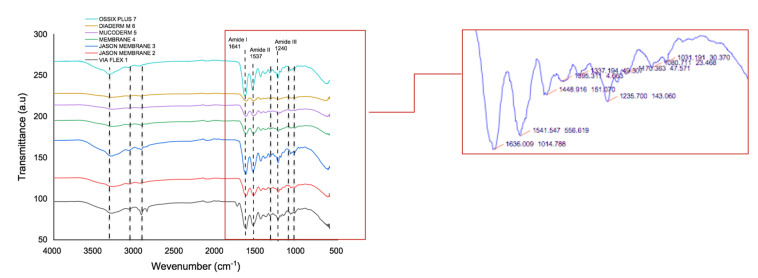
Characteristic vibrations of the chemical functional groups present in collagen by FT-IR.

The graph in Figure 4 shows the FTIR spectra of all the samples analyzed, revealing signals characteristic of collagen, the main structural component of the membranes. Specifically, Amide A, associated with N-H stretching in the range of 3400–3500 cm^−1^, and Amide B in the range of 3000–2950 cm^−1^, were identified. Additionally, bands corresponding to C-H stretching were observed between 2800 and 2900 cm^−1^.

The presence of collagen was further confirmed by the identification of Amide I (1641 cm^−1^), primarily associated with C=O bonds; Amide II (1537 cm^−1^); and Amide III (1240 cm^−1^), attributed to N-H stretching, tertiary amines, cyclic amines, and C-N bond stretching. A band at 1337.96 cm^−1^ corresponding to C-N stretching was also detected. Finally, between 1000 and 1100 cm^−1^, bands characteristic of C-O-H, C-O, and C-O-C deformations—typical of carbohydrates and carboxylic acids—were observed.

## 4. Discussion

Collagen is a natural polymer that has attracted a great amount of research, and it has been widely used in the clinical setting because of its properties, which make it suitable for GBR procedures [5,33]. Membranes made from collagen have been shown to provide a familiar environment for osteoblasts, which facilitates their adhesion and growth [5]. In addition to the advantages of a one-step procedure, collagen membranes clinically accelerate wound stabilization and initial defect closure [34]. This is because it is the predominant component of the extracellular matrix [35].

Collagen membranes can be derived from various tissue sources, such as skin, pericardium, peritoneum, and tendons. Studies have demonstrated that their regenerative properties vary depending on the tissue origin [30,32]. Collagen from different sources exhibits distinct morphological characteristics, which significantly influence the cellular response and the degradation pattern of collagen membranes in vivo [5]. Therefore, analyzing and comparing the properties of collagen membranes from different tissue origins is crucial for advancing future research in this area. This study examined porcine collagen membranes of different origins using infrared spectroscopy, thermal analysis, and differential scanning calorimetry (DSC). The morphological and morphometric characteristics were analyzed through scanning electron microscopy (SEM) and energy-dispersive X-ray spectroscopy (EDX). Additionally, the absorption capacity of the membranes was evaluated using an analytical digital balance. The results provide valuable insights into the fundamental differences among porcine collagen membranes from different tissue sources.

Collagen membranes are composed of various proteins, including fibrillar collagens, non-fibrillar collagens, and leucine-rich repeat proteoglycans, as well as a small number of structural proteins such as vimentin, actin-based microfilaments, annexins, tubulins, and histones [36]. The present study, using energy-dispersive X-ray spectroscopy (EDX), demonstrated that the composition of membranes from different tissue sources did not vary significantly. Specifically, the weight percentages of carbon, nitrogen, and oxygen were similar across membranes derived from skin, tendons, dermis, pericardium, and peritoneum. This finding was further corroborated by Fourier transform infrared (FT-IR) spectroscopy. The IR spectra revealed functional groups characteristic of collagen, including amides A, B, I, II, and III [37]. The characteristic vibrations corresponding to these functional groups were consistently identified across all samples, suggesting that the structural properties of collagen in membranes from different origins are highly similar.

The Amide I band, located at 1641 cm^−1^ in all membranes, is primarily attributed to the C = O (carbonyl) stretching vibration in collagen [38]. This band is critical for confirming the presence of collagen, particularly the triple helix structure. Absorption in this region indicates the structural stability of collagen and serves as a direct marker of protein conformation [39,40]. The Amide II band at 1537 cm^−1^ is associated with N-H stretching and C-N deformation, reflecting the peptide bonds within collagen. This band reinforces the presence of collagen and indicates the presence of peptide bonds that connect amino acids in the protein [41]. The Amide III band, located at 1240 cm^−1^, is related to N-H stretching and C-N bond stretching [38]. This band further supports the identification of collagen, as previously reported in the literature [38,41,42].

Thermogravimetric analysis (TGA) and differential scanning calorimetry (DSC) showed that the collagen membranes studied have similar composition and thermal behavior, with well-defined thermal transitions corresponding to the loss of structural water and collagen decomposition at elevated temperatures. This is consistent with the fact that these tissues predominantly contain type I collagen, a protein that constitutes 90% of the human body [43]. Therefore, purifying these tissues is a demanding process, which facilitates the production of high-quality, pure collagen membranes.

Although the membranes presented a similar chemical composition, their morphological properties differed. The morphology adopted by the collagen membranes is consistent with the morphology of the tissue of origin. In mammals, the skin consists of three layers of epidermis, dermis, and hypodermis [44,45,46]. There are membranes for GBR created from skin (Diaderm) and dermis (mucoderm). However, in membranes made from skin, purification procedures usually eliminate the epidermis and hypodermis [5]. The dermis contains 60–70% collagen, and its fibers are distributed anisotropically, arranged in a loose network, which, in combination with a large amount of water, fills the spaces between the collagen fibers [5]. The purification of this tissue makes it possible to obtain a membrane with random fibers that form a highly porous, rough surface. On the other hand, the tendon is a connective tissue composed mainly of collagen fibers [47,48]. These collagen fibers tend to be arranged in a dense and organized structure, with fewer interstitial spaces between fibers than other tissues such as skin [5,43]. Therefore, the structure of membranes created from tendon is characterized by compactness and low porosity. Membranes made from pericardium are widely used in clinical practice. This is because they exhibit excellent multidirectional tear strength due to the natural arrangement of collagen similar to a cross-linked honeycomb [47]. Finally, membranes created from peritoneum are characterized by a compact longitudinal fiber arrangement [1]. Therefore, it is important to consider the tissue origin of collagen membranes as they differ in their surface topography and porosity, which impacts soft tissue response and GBR [49].

Previous studies suggest that the morphological properties of polymeric membranes are important factors as they determine their bioresorbability and osteoregenerative effect in vivo [49]. Pore size is a morphological characteristic that affects the degree of bone regeneration, as it is closely related to tissue occlusivity and greatly influences soft tissue cell invasion [9]. Despite many studies devoted to the role of membrane permeability and porosity, there are conflicting results regarding membrane porosity [7]. This is because it is essential to hinder soft tissue invasion and facilitate the diffusion of fluids, oxygen, nutrients, and bioactive substances for cell growth, which is vital for bone and soft tissue regeneration [9,10,50,51]. This study provides evidence of significant differences in the pore size of membranes of different origins. The tendon, skin, and peritoneum membranes showed the smallest pore size. The reported size would prevent the passage of fibroblasts (10–15 microns) to the bone region [52]. In contrast, the pericardium membrane had the largest pore size. The pericardium, the membrane surrounding the heart, has a structure and composition that can naturally make it more porous than tendon or skin. In addition, the preparation procedures for collagen membranes for GBR can affect the structure, degradation, and final porosity of the material [1,39,53]. Therefore, pore size varies among membranes of different origins, ranging from nearly solid to macroporous [35,53].

Additionally, this study determined that the membrane with a larger pore size, made from pericardium, allowed the absorption of a greater amount of liquid (PBS). This is relevant, as collagen membranes have been shown to adsorb active factors released from bone and cells as a molecular mechanism contributing to bone regeneration [1,54]. Therefore, the ability to absorb fluid has been linked to increased hemostatic performance and enhanced tissue regeneration [55,56]. In addition, nanoscale studies have shown that during the first contact of a biomaterial with blood, proteins are adsorbed on the surface of the biomaterial. The adsorbed proteins create an interface that regulates immune cell functionality [57]. Therefore, the pore size and absorbance capacity of the membranes are important factors to consider for GBR. However, more systematic investigations are needed to address the porosity percentage, interconnectivity, and pore size required by a GBR membrane in the bone healing mechanism in the treated defect.

Elgali et al. determined that collagen membranes have different structures and thicknesses depending on the collagen source and the membrane manufacturing method [9]. The present study corroborated Elgali et al. in that the membrane pore size, morphology, and thickness were variable in the different sources [9]. The results obtained show that there could be an inversely proportional relationship between collagen membrane thickness and absorption. It was revealed that the thicker collagen membrane created from dermis presented lower absorbance. In contrast, the thinner membrane made from pericardium showed higher absorbance. These results coincide with those reported by Caballaré Serrano [1]. The less thick collagen membrane (0.15 mm), manufactured from pericardium, had the highest absorption capacity, and the collagen membranes of greatest thickness (0.9 mm), created from dermis, presented the lowest absorption capacity [1]. However, in materials of synthetic origin, it has been shown that a smaller membrane thickness is accompanied by a lower absorption capacity [1]. Therefore, the thickness of the membrane and the tissue/biomaterial of origin are determining factors in the absorption capacity of membranes [58].

## 5. Conclusions

This study demonstrates that although collagen membranes share a similar basic composition, they present differences in morphology, structure, and absorption properties that can significantly influence their physicochemical and biological performance. These variations should be considered when selecting the most suitable membrane for guided bone regeneration procedures, as certain properties may promote tissue integration and healing. Therefore, it is important to consider these differences when selecting the most appropriate collagen membrane for a specific tissue regeneration procedure.

## Figures and Tables

**Table 1:** Characteristics of the collagen membranes used in this study.

Membrane	Origin	Characteristics	Biodegradation	Batch Number	Expiry Date
**Diaderm M**	Skin	Atelocollagen. Biodegradable, thin, flexible, easy to handle, and biocompatible.	YES	BB042106GM	20 April 2025
**Jason membrane**	Pericardium	Membrane-type guided tissue regeneration (GTR). Natural three-dimensional collagen membrane. Easy to apply, approximately 0.4 mm thick, with a rough and porous structure.	YES	22AA51050	January 2025-
**mucoderm**	Dermis	Thickness varies between 0.8 and 5.0 mm	YES	21X12-14	December 2026
**OSSiX plus**	Tendons	Cross-linked, resorbable collagen membrane for guided hard tissue regeneration, easy to handle, biocompatible, and degradation-resistant. Pore size small enough to occlude gingival cells but large enough to allow passage of fluid, nutrients, and plasma proteins	YES	OP1500511	14 March 2025
**Via Flex**	Peritoneum	Meso Dental Membrane is a non-pyrogenic porcine-derived dental barrier membrane and is resorbable.	YES	7009236	28 February 2025

**Table 2:** Mean and standard deviation of pore size in the different membrane samples analyzed.

Diaderm M	Jason Membrane	Mucoderm	Ossix Plus	Via Flex
1.44 ± 1.24 µm	78.90 ± 75.89 µm	64.89 ± 13.15 µm	0.46 ± 0.1 µm	9.02 ± 3.70 µm

**Table 3:** Mean and standard deviation of membrane thickness in the different membrane samples analyzed.

Diaderm M	Jason Membrane	Mucoderm	OSSiX Plus	Via Flex
129 ± 20.40 µm	97.86 ± 45.66 µm	1352 ± 120 µm	246.17 ± 39.36 µm	311.83 ± 219.19 µm

**Table 4:** Mean and standard deviation of weight percentage of chemical composition (wt.%) in the different membrane samples analyzed.

Membrane	C	N	O
Diaderm M	78.79 ± 0.11	11.10 ± 0.02	10.11 ± 0.10
Jason membrane	78.66 ± 0.60	10.78 ± 0.13	10.39 ± 0.51
mucoderm	81.85 ± 3.60	7.83 ± 0.10	10.32 ± 0.35
OSSiX plus	77.45 ± 1.83	10.03 ± 0.65	11.37 ± 1.33
Via Flex	81.75 ± 0.50	9.70 ± 0.25	8.55 ± 0.24

C = carbon; N = nitrogen; O = oxygen.

**Table 5:** Absorption ability of the collagen membranes.

Absorption (x-Fold Fncrease)						
	2′	4′	6′	8′	10′	12′
Diaderm M	2.52	3.12	3.48	3.56	3.56	3.56
Jason membrane	6.07	7.29	10.23	10.24	10.24	10.24
mucoderm	3.89	4.01	4.07	4.09	4.09	4.09
OSSiX plus	4.48	4.80	5.31	5.32	5.92	5.92
Vía Flex	4.26	4.49	4.60	4.99	5.76	5.76

**Table 6:** Temperature transitions of the collagen membranes.

Membranas	T1	T2
Diaderm M	~81	205.03
Jason membrane		211.63
mucoderm		201.91
OSSiX plus		205.81
Vía Flex		208.37

**Table 7:** Proximal analysis of the collagen membranes.

Membranas	Humedad (%)	Cenizas (%)	Volatiles y Carbono Fijo (%)
Diaderm M	10.26	23.37	66.23
Jason membrane	12.95	21.75	65.19
mucoderm	13.37	22.23	64.36
OSSiX plus	13.17	21.94	64.64
Vía Flex	9.97	16.76	73.22

## Data Availability

Data are contained within the article.

## References

[1] Caballé-Serrano J., Munar-Frau A., Delgado L., Pérez R., Hernández –Alfaro F. (2019). Physicochemical characterization of barrier membranes for bone regeneration. J. Mech. Behav. Biomed. Mater..

[2] Lee S.W., Kim S.G. (2014). Membranes for the guided bone regeneration. Maxillofac. Plast. Reconstr. Surg..

[3] Liu J., Kerns D.G. (2014). Mechanisms of guided bone regeneration: A review. Open Dent. J..

[4] Hoornaert A., d’Arros C., Heymann M.F., Layrolle P. (2016). Biocompatibility, resorption and biofunctionality of a new synthetic biodegradable membrane for guided bone regeneration. Biomed. Mater..

[5] Ren Y., Fan L., Alkildani S., Liu L., Emmert S., Najman S., Rimashevskiy D., Schnettler R., Jung O., Xiong X. (2022). Barrier Membranes for Guided Bone Regeneration (GBR): A focus on recent advances in collagen membranes. Int. J. Mol. Sci..

[6] Kim Y.K., Ku J.K. (2020). Guided bone regeneration. J. Korean Assoc. Oral Maxillofac. Surg..

[7] Sanz M., Dahlin C., Apatzidou D., Artzi Z., Bozic D., Calciolari E., De Bruyn H., Dommisch H., Donos N., Eickholz P. (2019). Biomaterials and regenerative technologies used in bone regeneration in the craniomaxillofacial region: Consensus report of group 2 of the 15th European Workshop on Periodontology on Bone Regeneration. J. Clin. Periodontol..

[8] Omar O., Elgali I., Dahlin C., Thomsen P. (2019). Barrier membranes: More than the barrier effect?. J. Clin. Periodontol..

[9] Elgali I., Omar O., Dahlin C., Thomsen P. (2017). Guided bone regeneration: Materials and biological mechanisms revisited. Eur. J. Oral Sci..

[10] Polimeni G., Koo K.T., Qahash M., Xiropaidis A.V., Albandar J.M., Wikesjö U.M. (2004). Prognostic factors for alveolar regeneration: Effect of tissue occlusion on alveolar bone regeneration with guided tissue regeneration. J. Clin. Periodontol..

[11] Calciolari E., Ravanetti F., Strange A., Mardas N., Bozec L., Cacchioli A., Kostomitsopoulos N., Donos N. (2018). Degradation pattern of a porcine collagen membrane in an in vivo model of guided bone regeneration. J. Periodontal Res..

[12] Bilal B., Niazi R., Nadeem S., Farid M.A., Nazir M.S., Akhter T., Javed M., Mohyuddin A., Rauf A., Ali Z. (2022). Fabrication of Guided Tissue Regeneration Membrane Using Lignin-Mediated ZnO Nanoparticles in Biopolymer Matrix for Antimicrobial Activity. Front. Chem..

[13] Jo Y.-Y., Oh J.-H. (2018). New Resorbable Membrane materials for guided bone regeneration. Appl. Sci..

[14] Gao Y., Wang S., Shi B., Wang Y., Chen Y., Wang X., Lee E.-S., Jiang H.-B. (2022). Advances in modification methods based on biodegradable membranes in guided bone/tissue regeneration: A review. Polymers.

[15] Sbricoli L., Guazzo R., Annunziata M., Gobbato L., Bressan E., Nastri L. (2020). Selection of collagen membranes for bone regeneration: A literature review. Materials.

[16] Gottlow J. (1993). Guided tissue regeneration using bioresorbable and non-resorbable devices: Initial healing and long-term results. J. Periodontol..

[17] Murphy K.G. (1995). Postoperative healing complications associated with Gore-Tex Periodontal Material. Part I. Incidence and characterization. Int. J. Periodontics Restor. Dent..

[18] Mizraji G., Davidzohn A., Gursoy M., Gursoy U., Shapira L., Wilensky A. (2000). Membrane barriers for guided bone regeneration: An overview of available biomaterials. Periodontology.

[19] Nair A., Chuang S.C., Lin Y.S., Chen C.H., Fang T.C., Chiu H.C., Lien C.H., Chen S.J. (2022). Characterization of collagen response to bone fracture healing using polarization-SHG. Sci. Rep..

[20] Davison-Kotler E., Marshall W.S., García-Gareta E. (2019). Sources of Collagen for Biomaterials in Skin Wound Healing. Bioengineering.

[21] Amirrah I.N., Lokanathan Y., Zulkiflee I., Wee M.F.M.R., Motta A., Fauzi M.B. (2022). A Comprehensive Review on Collagen Type I Development of Biomaterials for Tissue Engineering: From Biosynthesis to Bioscaffold. Biomedicines.

[22] Santos M.H., Silva R.M., Dumont V.C., Neves J.S., Mansur H.S., Heneine L.G. (2013). Extraction and characterization of highly purified collagen from bovine pericardium for potential bioengineering applications. Mater. Sci. Eng. C Mater. Biol. Appl..

[23] Vaage J., Lindblad W.J. (1990). Production of collagen type I by mouse peritoneal macrophages. J. Leukoc. Biol..

[24] Feng Y., Shi Y., Tian Y., Yang Y., Wang J., Guo H., Banitaba S.N., Khademolqorani S., Li J. (2023). The Collagen-Based Scaffolds for Bone Regeneration: A Journey through Electrospun Composites Integrated with Organic and Inorganic Additives. Processes.

[25] Fan L., Ren Y., Emmert S., Vučković I., Stojanovic S., Najman S., Schnettler R., Barbeck M., Schenke-Layland K., Xiong X. (2023). The Use of Collagen-Based Materials in Bone Tissue Engineering. Int. J. Mol. Sci..

[26] Dimitriou R., Mataliotakis G.I., Calori G.M., Giannoudis P.V. (2012). The role of barrier membranes for guided bone regeneration and restoration of large bone defects: Current experimental and clinical evidence. BMC Med..

[27] Aprile P., Letourneur D., Simon-Yarza T. (2020). Membranes for guided bone regeneration: A road from bench to bedside. Adv. Healthc. Mater..

[28] Patino M.G., Neiders M.E., Andreanna S., Noble B., Cohen R.E. (2002). Collagen: An overview. Implant Dent..

[29] Patino M.G., Neiders M.E., Andreana S., Noble B., Cohen R.E. (2002). Collagen as an implantable material in medicine and dentistry. J. Oral Implantol..

[30] Radenković M., Alkildani S., Stoewe I., Bielenstein J., Sundag B., Bellmann O., Jung O., Najman S., Stojanović S., Barbeck M. (2021). Comparative In Vivo Analysis of the Integration Behavior and Immune Response of Collagen-Based Dental Barrier Membranes for Guided Bone Regeneration (GBR). Membranes.

[31] Hirata H.H., Munhoz M.A., Plepis A.M., Martins V.C., Santos G.R., Galdeano E.A., Cunha M.R. (2015). Feasibility study of collagen membranes derived from bovine pericardium and intestinal serosa for the repair of cranial defects in ovariectomised rats. Injury.

[32] Shi X., Li X., Tian Y., Qu X., Zhai S., Liu Y., Jia W., Cui Y., Chu S. (2023). Physical, mechanical, and biological properties of collagen membranes for guided bone regeneration: A comparative in vitro study. BMC Oral Health.

[33] Wang Y., Wang Z., Dong Y. (2023). Collagen-based biomaterials for tissue engineering. ACS Biomater. Sci. Eng..

[34] Kumari C.B.N., Ramakrishnan T., Devadoss P., Vijayalakshmi R., Alzahrani K.J., Almasri M.A., Al-Ahmari M.M., Al Dira H.S., Suhluli M., Bhati A.K. (2021). Use of collagen membrane in the treatment of periodontal defects distal to mandibular second molars following surgical removal of impacted mandibular third molars: A comparative clinical study. Biology.

[35] Ricard-Blum S. (2011). The collagen family. Cold Spring Harb. Perspect. Biol..

[36] Lee J.S., Mitulović G., Panahipour L., Gruber R. (2020). Proteomic analysis of porcine-derived collagen membrane and matrix. Materials.

[37] Xiao H., Guoping C., Liu M. (2007). Hydroxyl radical induced structural changes of collagen. Spectroscopy.

[38] León-Mancilla B.H., Araiza-Téllez M.A., Flores-Flores J.O., Piña-Barba M.C. (2016). Physico-chemical characterization of collagen scaffolds for tissue engineering. J. Appl. Res. Technol..

[39] Payne K.J., Veis A. (1988). Fourier transform ir spectroscopy of collagen and gelatin solutions: Deconvolution of the amide I band for conformational studies. Biopolímeros.

[40] Lam R.S., Metzler R.A., Gilbert P.U., Beniash E. (2012). Anisotropy of chemical bonds in collagen molecules studied by X-ray absorption near-edge structure (XANES) spectroscopy. ACS Chem. Biol..

[41] Wang J., Qu Y., Chen C., Sun J., Pan H., Shao C., Tang R., Gu X. (2019). Fabrication of collagen membranes with different intrafibrillar mineralization degree as a potential use for GBR. Mater. Sci. Eng. C Mater. Biol. Appl..

[42] Valencia-Llano C.H., López-Tenorio D., Grande-Tovar C.D. (2022). Biocompatibility Assessment of Two Commercial Bone Xenografts by In Vitro and In Vivo Methods. Polymers.

[43] Naomi R., Ridzuan P.M., Bahari H. (2021). Current insights into collagen type I. Polymers.

[44] Meyer M. (2019). Processing of collagen based biomaterials and the resulting materials properties. Biomed. Eng. Online.

[45] Terzi A., Gallo N., Bettini S., Sibillano T., Altamura D., Madaghiele M., De Caro L., Valli L., Salvatore L., Sannino A. (2020). Sub- and supramolecular x-ray characterization of engineered tissues from equine tendon, bovine dermis, and fish skin type-I collagen. Macromol. Biosci..

[46] Reilly D.M., Lozano J. (2021). Skin collagen through the lifestages: Importance for skin health and beauty. Plast. Aesthetic Res..

[47] Ratiu C., Brocks M., Costea T., Moldovan L., Cavalu S. (2019). PRGF-modified collagen membranes for guided bone regeneration: Spectroscopic, microscopic and nano-mechanical investigations. Appl. Sci..

[48] Franchi M., Trirè A., Quaranta M., Orsini E., Ottani V. (2007). Collagen structure of tendon relates to function. Sci. World J..

[49] de Santana R.B., de Mattos C.M., Francischone C.E., Van Dyke T. (2010). Superficial topography and porosity of an absorbable barrier membrane impacts soft tissue response in guided bone regeneration. J. Periodontol..

[50] Gutta R., Baker R.A., Bartolucci A.A., Louis P.J. (2009). Barrier membranes used for ridge augmentation: Is there an optimal pore size?. J. Oral Maxillofac. Surg..

[51] Oh S.H., Kim J.H., Kim J.M., Lee J.H. (2006). Asymmetrically porous PLGA/Pluronic F127 membrane for effective guided bone regeneration. J. Biomater. Sci. Polym. Ed..

[52] Michael H.R., Edward J.R., Lynn J.R. (1989). Histology: A Text and Atlas, 2nd edi..

[53] Caballé-Serrano J., Munar-Frau A., Ortiz-Puigpelat O., Soto-Penaloza D., Peñarrocha M., Hernández-Alfaro F. (2018). On the search of the ideal barrier membrane for guided bone regeneration. J. Clin. Exp. Dent..

[54] Caballé-Serrano J., Sawada K., Miron R.J., Bosshardt D.D., Buser D., Gruber R. (2017). Collagen barrier membranes adsorb growth factors liberated from autogenous bone chips. Clin. Oral Implants Res..

[55] Zhang Y., Wang C., Jiang W., Zuo W., Han G. (2017). Influence of stage cooling method on pore architecture of biomimetic alginate scaffolds. Sci. Rep..

[56] Zhang D., Wu X., Chen J., Lin K. (2017). The development of collagen based composite scaffolds for bone regeneration. Bioact. Mater..

[57] Gallagher W.M., Lynch I., Allen L.T., Miller I., Penney S.C., O’Connor D.P., Pennington S., Keenan A.K., Dawson K.A. (2006). Molecular basis of cell-biomaterial interaction: Insights gained from transcriptomic and proteomic studies. Biomaterials.

[58] Bubalo M., Lazić Z., Matić S., Tatić Z., Milović R., Curcin A.P., Djurdjević D., Loncarević S. (2012). The impact of thickness of resorbable membrane of human origin on the ossification of bone defects: A pathohistologic study. Vojnosanit. Pregl..

